# Changes in White-Matter Connectivity in Late Second Language Learners: Evidence from Diffusion Tensor Imaging

**DOI:** 10.3389/fpsyg.2017.02040

**Published:** 2017-11-21

**Authors:** Eleonora Rossi, Hu Cheng, Judith F. Kroll, Michele T. Diaz, Sharlene D. Newman

**Affiliations:** ^1^Department of Psychology and Sociology, California State Polytechnic University, Pomona, Pomona, CA, United States; ^2^Department of Psychology, University of California, Riverside, Riverside, CA, United States; ^3^Department of Psychology, Indiana University, Bloomington, IN, United States; ^4^Department of Psychology, Pennsylvania State University, University Park, PA, United States

**Keywords:** diffusion tensor imaging, bilingualism, second language learning, neuroplasticity, age of acquisition

## Abstract

Morphological brain changes as a consequence of new learning have been widely established. Learning a second language (L2) is one such experience that can lead to rapid structural neural changes. However, still relatively little is known about how levels of proficiency in the L2 and the age at which the L2 is learned influence brain neuroplasticity. The goal of this study is to provide novel evidence for the effect of bilingualism on white matter structure in relatively proficient but late L2 learners who acquired the second language after early childhood. Overall, the results demonstrate a significant effect on white matter fractional anisotropy (FA) as a function of L2 learning. Higher FA values were found in a broad white matter network including the anterior thalamic radiation (ATR), the inferior fronto-occipital fasciculus (IFOF), the Uncinate Fasciculus (UF), and the inferior longitudinal fasciculus (ILF). Moreover, FA values were correlated with age of L2 acquisition, suggesting that learning an L2, even past childhood, induces neural changes. Finally, these results provide some initial evidence that variability in the age of L2 acquisition has important consequences for neural plasticity.

Learning a second language (L2) after a putative critical period for language learning (Long, 1990; Birdsong, 1999) is notably difficult, especially when the native language (L1) and the L2 are linguistically different. Past research on late L2 attainment suggesting mixed outcomes has been interpreted in different ways. One perspective proposes that late L2 representation and processing is hard-wired by maturational constraints and is fundamentally different than native language processing, especially when the grammatical structures of the two languages differ (e.g., Johnson and Newport, 1991; Weber-Fox and Neville, 1996; MacWhinney, 2005; Clahsen and Felser, 2006; Sabourin et al., 2006; Sabourin and Stowe, 2008). In contrast, processing-based accounts of L2 acquisition posit that native-like processing is possible for individuals who acquire an L2 after childhood, with some late learners acquiring a high level of L2 proficiency (e.g., McDonald, 2000; Birdsong and Molis, 2001; McLaughlin et al., 2010; Coughlin and Tremblay, 2012; Rossi et al., 2014). Other studies have shown that proficient late L2 speakers are also able to exploit cognitive resources that are central for on-line language processing (e.g., Hopp, 2010, 2014; Linck et al., 2014). Moreover, near native-like L2 processing has been correlated with immersion in the L2 environment, even when the experience was brief (Linck et al., 2009), suggesting that L2 processing is sensitive to variability in the frequency of usage and characteristics of L2 exposure (Ellis and Ogden, 2017).

The long-standing question of the nature of L2 representation and processing has also been extended to the realm of its neural underpinnings and has fueled a wealth of functional neuroimaging research with the goal of investigating if the functional neural networks underlying L2 processing are similar to the ones observed during native language processing, and to ask whether variables such as proficiency and age of acquisition (AoA) modulate the recruitment of those networks (see Li et al., 2014; García-Pentón et al., 2015; Luk and Pliatsikas, 2016 for recent reviews). Overall, functional evidence suggests that both languages are supported by similar cortical substrates even when the L2 is acquired relatively later in life, and that the recruitment of those networks is influenced by AoA (Perani et al., 1996, 1998; Wartenburger et al., 2003; Perani and Abutalebi, 2005) and also proficiency levels (Perani et al., 1998; Abutalebi et al., 2001). Very recently however, Xu et al. (2017) used multivariate pattern analysis (MVPA) to challenge the traditional single cortical mechanism hypothesis, proposing instead that the two languages might share the same neural substrate but may critically be supported by functionally independent networks. Critically, bilingualism and L2 learning lead also to the reorganization of neural areas that are not specifically related to language processing, but are involved in domain-general executive functions (Crinion et al., 2006; Li et al., 2014; Bialystok, 2017). The recruitment of domain-general brain areas such as the anterior cingulate cortex (Abutalebi et al., 2012), and subcortical structures such as the caudate (Abutalebi et al., 2008; Branzi et al., 2015) have been linked to mechanisms involved in language regulation, activation, and selection that are necessary in the face of ubiquitous co-activation of both languages, even when bilinguals intend to speak one language alone (e.g., Costa, 2005; Kroll et al., 2006). One prominent account proposes that for bilinguals to be able to successfully speak and control their languages, they engage a dynamic domain general neural network involving cortical and subcortical brain structures that allows them to resolve language competition to successfully select the intended language (Green, 1998; Abutalebi and Green, 2007; Green and Abutalebi, 2013).

Despite the wealth of research on the functional underpinnings of L2 processing, fewer studies have investigated the extent to which learning an L2 promotes *structural* brain changes. Early seminal research on neuroplasticity in animal models (Rosenzweig et al., 1962; Bennett et al., 1964; Diamond et al., 1964) demonstrated that the brain is not an immutable organ, but is pliable, and influenced by enriched environmental conditions and different task demands. Similarly, research on structural and morphological brain changes in the human brain have revealed that the brain is highly malleable and changes as a function of different types of skill learning. Neuroplastic changes in gray matter (GM) and white matter (WM) have been demonstrated across a vast array of skill and motor learning tasks (Draganski et al., 2004; Bengtsson et al., 2005), visual memory (Maguire et al., 2000), music practice (Skare et al., 2005), and even higher-level meditation practices (Hernández et al., 2016).

Crucially, learning and juggling two languages constitute a prime example of new skill acquisition, especially when the L2 is learned past childhood and its acquisition is largely dependent on explicit learning mechanisms (Ullman, 2016). It is possible that *late* L2 learning in particular might be considered the perfect testbed to examine the effect of neuroplastic changes as a consequence of language learning. In fact, actively learning and mastering an L2, especially later in life might involve re-training and restructuring of a number of neural structures related to L2 language production, articulation, and language comprehension, potentially leading to greater neural changes especially during the most active learning phases (Xiang et al., 2015). Although, neuroplasticity may decrease across the life-span (Kennedy and Raz, 2009) resulting in smaller detectable changes after childhood, we hypothesize that adolescent or adult L2 language learning may be a sufficiently challenging task to elicit neural changes even in the face of reduced neuroplasticity. This idea resonates with the literature on desirable difficulties in learning, which proposes that L2 language learning and use is inherently taxing for the cognitive and neural system, but it is exactly that inherent difficulty that will produce long-term positive consequences for domain-general functions (Bjork and Kroll, 2015).

Evidence in favor of the neuroplastic effects of bilingualism is growing (Costa and Sebastián-Gallés, 2014). In a seminal study, Mechelli and colleagues demonstrated that bilinguals have greater GM density in the left inferior parietal lobule than monolingual controls (Mechelli et al., 2004; Della Rosa et al., 2013), and that the effect is modulated by AoA and proficiency, with earlier exposure to the L2 and higher L2 proficiency being positively correlated with higher GM. Similarly, greater GM density in the left inferior parietal gyrus (LIPG) has been reported in older bilingual adults (Abutalebi et al., 2015a), however with no correlations with AoA or proficiency. Differences between bilinguals and monolinguals in GM surface area and cortical thickness have also been shown in non-language related areas, with greater GM in the anterior cingulate cortex (Abutalebi et al., 2012; Felton et al., 2017). Finally, greater GM volume in bilinguals has been documented in several other areas, including the caudate nucleus (e.g., Grogan et al., 2012; Zou et al., 2012b), and putamen (Abutalebi et al., 2013a) which are subcortical areas that are important for language selection and control, both in non-pathological bilingual language processing (e.g., Abutalebi et al., 2007), and in the face of pathology (Green and Abutalebi, 2008). Increases in GM density in left IFG have also been found after a 5-month period of immersed L2 learning, suggesting again that L2 learning promotes fast neural restructuring (Stein et al., 2012).

Research on the neural changes promoted by bilingualism and L2 learning has also examined effects on white matter connectivity. To date however, even though the literature is rapidly growing, the majority of the research has examined simultaneous or early bilinguals who acquired their two languages during early childhood. For example, a study comparing early bilingual children to sequential bilingual children (who learned the L2 at 3 years old) and monolingual children revealed that white matter microstructure (measured through fractional anisotropy, FA) in language-related bundles is positively modulated by bilingualism, and has provided evidence that the magnitude of the effect is dependent on AoA (Mohades et al., 2012, 2015). In these studies, Mohades and colleagues analyzed four WM tracts, including the left inferior frontal-occipital fasciculus (IFOF), the left arcuate fasciculus/superior longitudinal fasciculus (SLF), the WM bundle from the anterior part of the corpus callosum projecting to the orbital frontal cortex, and WM fibers from the anterior midbody of the corpus callosum to premotor and supplementary motor cortices. Their results showed that simultaneous bilinguals have higher FA in L-IFOF which is a ventral WM pathway that has been proposed to be central during spoken word recognition (Leclercq et al., 2010), and semantic processing (Duffau, 2008; Duffau et al., 2009; Martino et al., 2010). Mohades and colleagues also reported that sequential bilinguals had intermediate FA values between monolinguals and simultaneous bilinguals. They concluded that early bilingualism leads to neural adaptation in the human brain. In a follow up 2-year longitudinal study, Mohades et al. (2015) tracked simultaneous, and sequential bilingual children who were learning an L2. The results showed again higher FA values in IFOF for simultaneous bilinguals, but crucially sequential bilinguals showed an even greater change in IFOF over the course of the 2 years. The authors concluded that the degree of neural reshaping induced by bilingualism and L2 learning is partly dependent on AoA. Similar conclusions have been reported by Hämäläinen et al. (2017) who compared a group of early and sequential bilinguals. They analyzed mean FA, mean and radial diffusivity (MD and RD), and found that early bilingualism led to higher WM in the arcuate fasciculus, while sequential bilinguals showed greater WM connectivity in bilateral Inferior fronto-occipital fasciculus (IFOF), suggesting that different ages of L2 acquisition might determine what WM tracts might be shaped by language experience. Recent data has also revealed separate WM structural networks depending on different AoA but also proficiency levels, suggesting that brain changes might be differentially shaped by these two factors (Nichols and Joanisse, 2016). In sum, research on WM changes in early bilinguals has demonstrated that acquiring two languages from early childhood, or even learning an L2 relatively early during childhood has neuroplastic effects on both language specific and domain general WM pathways (Kousaie et al., 2017). Importantly, studies of WM changes in early bilingualism highlight that it is misleading to characterize AoA as a discrete variable, but rather that AoA should be understood as a continuum, as even within early acquisition, differences in AoA are correlated with different quantitative and qualitative effects.

To date, relatively few studies have investigated white-matter reorganization following L2 acquisition past early childhood. One such study investigated differences in WM structures between monolinguals and young adults who were late L2 learners (Pliatsikas et al., 2015). The L2 learners (*n* = 20) had a variety of languages as their L1s and had acquired English past the age of 10, but were classified as highly proficient English speakers. Participants were tested in the UK, thus immersed in their L2 environment. The TBSS results revealed higher FA values for the L2 group in the corpus callosum, including the genu, the body, and the anterior part of the splenium. Higher FA values were also found in left and right IFOF, bilateral uncinate fasciculi, and superior longitudinal fasciculi, all WM tracts that have been found to be modulated in early bilinguals. However, no correlational effects were found with length of immersion in the L2. The authors concluded that there is an effect of bilingualism on WM structures even when the L2 is learned past childhood. Importantly, the observed WM structures that have been identified for late bilinguals are similar to the ones that have been reported to be shaped by bilingualism in older adults (Luk et al., 2011), and also in early bilinguals (Mohades et al., 2012), suggesting that neural structures undergo neuroplastic changes as a consequence of L2 learning and bilingualism irrespective of the age at which the L2 is acquired. Similar neuroplastic changes in white matter have been reported in Spanish-English bilinguals who immigrated to the US in adulthood (Kuhl et al., 2016), and who learned English later in life (mean age = 19.4 years; range = 4.5–28.5 years). These speakers were immersed in their L2 environment at testing, and were recruited from the general population. The results reveled higher FA values in the bilateral anterior thalamic radiation (ATR), a bundle of fibers that are part of the internal capsule, and carry nerve fibers between the thalamus and the prefrontal cortex. Additionally, Kuhl and colleagues found a positive correlation between FA values and years of immersion in the L2, and with speaking abilities, suggesting that the degree of neural restructuring in ATR was proportional to L2 language experience.

However, other studies have reported contrary results to the ones presented above. For example, Cummine and Boliek (2013) tested adult Chinese–English bilinguals (mean age, 24.2; L2 AoA before the age of 5) and 11 English monolinguals (mean age, 28.5). The results showed significant decrease in FA for bilinguals as compared to monolinguals in the right inferior frontal-occipital fasciculus (IFOF), and in the superior portion of the right anterior thalamic radiation, and bilaterally in the inferior portion. These results are also in line with other studies that did report minimal differences between bilingual children and monolinguals (e.g., Mohades et al., 2012).

A similar approach to studying the effects of late L2 learning on WM has been taken in studies that have asked the question of what neural changes occur when learning happens during a relatively short but intensive program of language training. Mamiya et al. (2016) recruited 44 native college-age Chinese speakers who were enrolled in a 16-day upper level English course. They collected structural scans (DTI) between the 11th day of the course and 8 days after the course ended. For those participants who were tested before the end of the course, results showed a significant cluster of activation in the right and left SLF, and a positive correlation with the number of days in the course. The same study also revealed a marginally negative correlation between FA values in the right SLF, and days passed after the end of the immersion course. The authors concluded that there is a relationship between the diffusion properties of the brain and the length of immersion, suggesting that changes in white matter are rapid. Similar results were reported by Schlegel et al. (2012) who tracked changes in white matter connectivity in a group of adult learners (mean age: 20.5) during a relatively longer 9-month intensive Chinese language course. Scans were acquired every month, and were compared to those of a comparable control group of individuals who did not attend any language course. Results showed a significant increase in FA values only for the learners in language-related WM tracts in the left hemisphere and in the genu of the corpus callosum, suggesting a strengthening of inter-hemispheric connections during L2 learning. Tract-based analysis also revealed that the learners group showed higher FA values in a number of tracts, some of which terminated in the left caudate nucleus which is implicated in language control (Green and Abutalebi, 2008), response selection (e.g., Branzi et al., 2015) and language-switching (Abutalebi et al., 2007). Similar results were found for a cohort of Japanese speakers who underwent 16 weeks of intensive English vocabulary training, while MRI scans were acquired before and after the training. Results revealed changes in right inferior frontal gyrus (IFG), arcuate fasciculus, and the pathways that connect IFG with the caudate nucleus (Hosoda et al., 2013). However, the observed WM changes reverted to baseline after 1 year, suggesting that neuroplastic changes might change depending on different demands. Similarly, Xiang et al. (2015) who tested a group of native German speakers who were enrolled in a 6-week intensive Dutch course while immersed in The Netherlands. Structural scans were acquired before and after the Dutch course. Results revealed a quick structural neural reorganization in connection with increasing L2 proficiency. A shift in hemispheric dominance was observed during early learning with greater FA values observed in the right arcuate fasciculus at early time points, which shifted back to the left with higher levels of L2 proficiency.

In sum, the recent literature suggests that WM pathways are modulated by L2 learning and bilingualism. However, evidence is still mixed regarding the relative contributions of proficiency and AoA, with data suggesting that that both proficiency and AoA play an important role in modulating those changes (e.g., Nichols and Joanisse, 2016; Hämäläinen et al., 2017). Regarding which WM pathways are most strongly impacted by bilingualism, a number of WM pathways have been highlighted as being frequently related to L2 learning and bilingualism. One such WM pathway is the SLF, a dorsal language network which connects posterior (superior temporal gyrus/Wernicke's area) and anterior (inferior frontal gyrus/ Broca's area) language cortices (Hickok and Poeppel, 2004, 2007). The IFOF instead, connects a ventral language network that includes Broca's area and posterior occipitotemporal regions, and also connects the anterior temporal lobe with the uncinate fasciculus (Anwander et al., 2007).

The goal of this study is to further examine the effects of L2 learning on WM in late L2 learners. To assess changes in WM we measured differences in fractional anisotropy (FA) in a group of monolingual speakers (*n* = 24) and a group of native English speaking late L2 learners of Spanish (*n* = 24) using tract-based spatial statistics (TBSS; Smith et al., 2006). FA can be used as an index of WM integrity, by reflecting the degree of anisotropy in water flow within the brain (Kunimatsu et al., 2004). If late L2 learning promotes neural adaptation, we should observe differences in FA values between the L2 learners and the monolinguals in WM tracts that have been previously found to be positively affected by bilingualism. For example, the left inferior frontal-occipital fasciculus (IFOF; Mohades et al., 2012, 2015; Pliatsikas et al., 2015; see García-Pentón et al., 2015 for an extensive review) which is closely connected to the left ILF (Wakana et al., 2007), the uncinate fasciculus, which has been implicated in naming (Catani and Mesulam, 2008; Papagno, 2011), and found to be modulated by bilingualism (Hosoda et al., 2013; Qi et al., 2015). Moreover, if late L2 learning also affects domain-general brain networks, effects should be seen in cortical-subcortical WM fibers that have been proposed to be utilized during bilingual language selection and control, such as fibers that connect the IFG with the caudate (Tan et al., 2011; Hosoda et al., 2013).

An additional goal of this study was to contribute to the growing literature on how proficiency and AoA, as well as factors related to L2 use and experience, such as length of immersion in an L2 environment, contribute to the observed neural restructuring.

## Materials and methods

### Participants

Twenty-four monolingual English speakers (15 females), and 25 (20 females) native English speaking, late learners of Spanish participated in the study (age range: 18–27). All participants were recruited from the student population at Pennsylvania State University and all were right-handed. They were screened for safety, and contraindications to MRI scanning, in accordance with IRB requirements. None of them reported having been diagnosed with any neurological or reading disorder and all had normal or corrected-to normal visual acuity. All participants completed a language history questionnaire to assess their language history and skills. The results from the questionnaire showed that English monolingual speakers had no or minimal knowledge of a second language. L2 Spanish speakers were native speakers of English who learned Spanish as their second language later in life (average L2 acquisition age: 12 years). They all reported to be dominant speakers of English. Participants rated their L1 and L2 language knowledge using a scale from 1 to 10 (1 being the lowest and 10 being the highest score) for oral comprehension, oral production, reading and writing. They were paid for their participation and all study procedures were approved by the IRB at Penn State University.

### Materials

**Figure 1:**
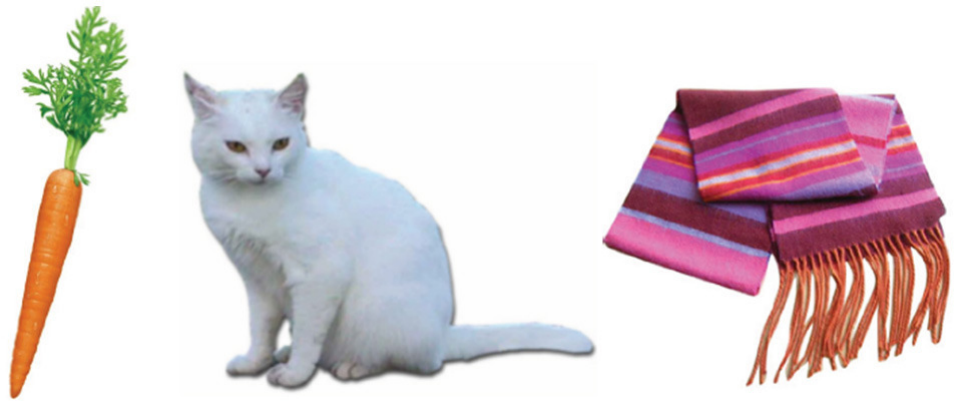
Examples of pictures used in the naming task.

As part of the testing battery, participants completed additional linguistic tasks that were designed to measure their proficiency in the L2 (Spanish). The language testing battery included a self-report language history questionnaire (reported in Appendix A) and a more objective grammar task. The primary task in the experiment was a picture naming task in English and Spanish that was part of an additional functional MRI study protocol that involved naming 6 runs of pictures (Rossi et al., in preparation). During this task, participants named a total of 144 items which were named in Spanish for the L2 learners group and in English for the English monolingual group. Stimuli consisted of images that were presented as line drawings, black and white photographs, or color photographs taken from 6 categories: animals, body parts, fruits, and vegetables, clothing, kitchen items, furniture. Within each category there were 16 items of each format for a total of 48 stimuli per category. Black and white and color photographs were identical except in color. The three formats were incorporated to allow for concept repetition, but minimize perceptually based priming. All images were 300 × 300 pixels and in bitmap image format. Across categories pictures were matched for frequency and imageability. All stimuli were presented using the Brain Logics MRI Digital Projection System, and experimental parameters were controlled via E-prime. Responses were recorded with an MR compatible microphone (Resonance Technologies, Northridge, CA). Examples of the stimuli are provided in Figure 1.

The grammar section of the Diploma de Español como Lengua Extranjera (DELE, Ministry of Education Culture Sport of Spain, 2006) was also administered to obtain an objective measure of grammatical knowledge in Spanish. Three sections of the DELE test were selected for this study. Participants completed the written text comprehension, the vocabulary and the grammar sections of the test. An example of the DELE test can be retrieved at: http://www.dele.org/. Finally, participants rated their L2 proficiency on a self-reported scale using a 0–10 scale, rating their language oral and written production and comprehension abilities. The full language history questionnaire is reported in Appendix A. The aggregate scores were calculated as follows: raw scores were standardized to z-scores and were summed together within each participant; then the resulting score was divided by the square root of the sum of the variances and covariances of all the subtests (Crocker and Algina, 1986; McMurray et al., 2010; Pivneva et al., 2012). These data are summarized in Table 1.

**Table 1:** Demographic and L2 language measures for the L2 Spanish learners.

**Gender**	**Average L2 self-rating**	**Picture naming accuracy Spanish**	**DELE score**	**L2 proficiency composite score**	**L2 Age of Acquisition (years)**	**Immersion time in L2 (months)**
F	7.0	0.861	0.86	1.750	11	8
M	8.75	0.583	0.74	1.756	13	6
F	10.0	0.750	0.72	2.810	16	24
F	7.25	0.403	0.48	−0.978	14	NA
F	9.0	0.819	0.82	3.282	12	6
F	6.75	0.528	0.42	−1.206	9	NA
M	7.50	0.819	0.68	1.551	12	6
F	6.25	0.347	0.44	−2.092	12	3
M	5.25	0.514	0.54	−1.348	12	4
F	7.00	0.625	0.6	0.211	12	NA
M	7.45	0.625	0.58	0.421	2	0
M	6.25	0.569	0.62	−0.333	12	0
F	6.00	0.625	0.42	−1.240	5	20
F	7.00	0.708	0.44	−0.363	13.5	0
M	7.13	0.458	0.52	−0.688	12	4
F	6.58	0.181	0.36	−2.642	8	0
F	6.25	0.278	0.28	−3.002	12	0
M	6.75	0.319	0.46	−1.512	13.5	0
F	8.5	0.806	0.56	1.529	19	22
M	7.5	0.986	0.64	1.685	14	5
M	8.5	0.722	0.58	1.408	13	1
F	6.75	0.283	0.3	−2.467	16	2
F	8.00	0.222	0.38	−1.231	13	0
F	8.25	0.250	0.45	−0.755	12	8.5
F	5.00	0.501	0.48	−1.785	14	20
Avg.	7.22	0.55	0.53	−0.20	12.08	6.34
*SD*	1.17	0.22	0.15	1.75	3.46	7.83

*NA: Data not provided in questionnaire*.

### Imaging pre-processing, procedures, and analysis

MRI scanning was completed on a Siemens 3.0 Tesla Magnetom Trio whole-body, human scanner (60 cm bore, 40 mT/m gradients, 200 T/m/s slew rate). An eight-channel head coil was used for Radio Frequency (RF) reception (Siemens Healthcare, Erlangen, Germany). Sagittal T-1 weighted localizer images were acquired and used to define a volume for high order shimming. The anterior and posterior commissures were identified for slice selection and shimming. A semi-automated high-order shimming program was used to ensure global field homogeneity. High-resolution structural images were acquired using a 3D MP-RAGE pulse sequence (TR = 1,400 ms; TE = 2.01 ms; TI = 900 ms; FOV = 25.6 cm^2^; flip angle = 9°; acceleration factor = 2; voxel size = 1 × 1 × 1 mm; 160 contiguous slices).

Diffusion Tensor Imaging (DTI) data were collected using the following parameters: TR/TE = 6,500/93 ms, FOV = 240 mm, matrix = 128 × 128, 48 slices, slice thickness = 3 mm with 20% gap, averages = 2. iPAT factor = 2, phase partial Fourier = 6/8, 20 diffusion directions, b = 1,000 s/mm^2^. DTI data were processed with FSL's FDT tool for eddy current correction and motion correction. Diffusion tensor was then computed using the tensor model to obtain FA values as inputs for TBSS analysis to examine the FA differences between monolinguals and English-Spanish bilinguals on the mean FA skeleton in FSL (Smith et al., 2004). The diffusion data were extracted first using BET (Smith, 2002). FA images were created by fitting a tensor model to the brain-extracted diffusion data using the FDT tool. FA's data were then are aligned into a common space using the non-linear registration tool FNIRT (Andersson et al., 2007a,b), which uses a b-spline representation of the registration warp field (Rueckert et al., 1999). Next, a mean FA image is created and thinned to create a mean FA skeleton, which represents the centers of all tracts common to the group. Each subject's aligned FA data is then projected onto this skeleton and permutation-based statistics of FA is conducted on all the voxels on the skeleton. In addition, regression analyses were performed to examine the relationship between FA and a number of behavioral and language usage measures, such as AoA, various measures of L2 proficiency (see below for details), and immersion in the L2.

## Results

**Figure 2:**
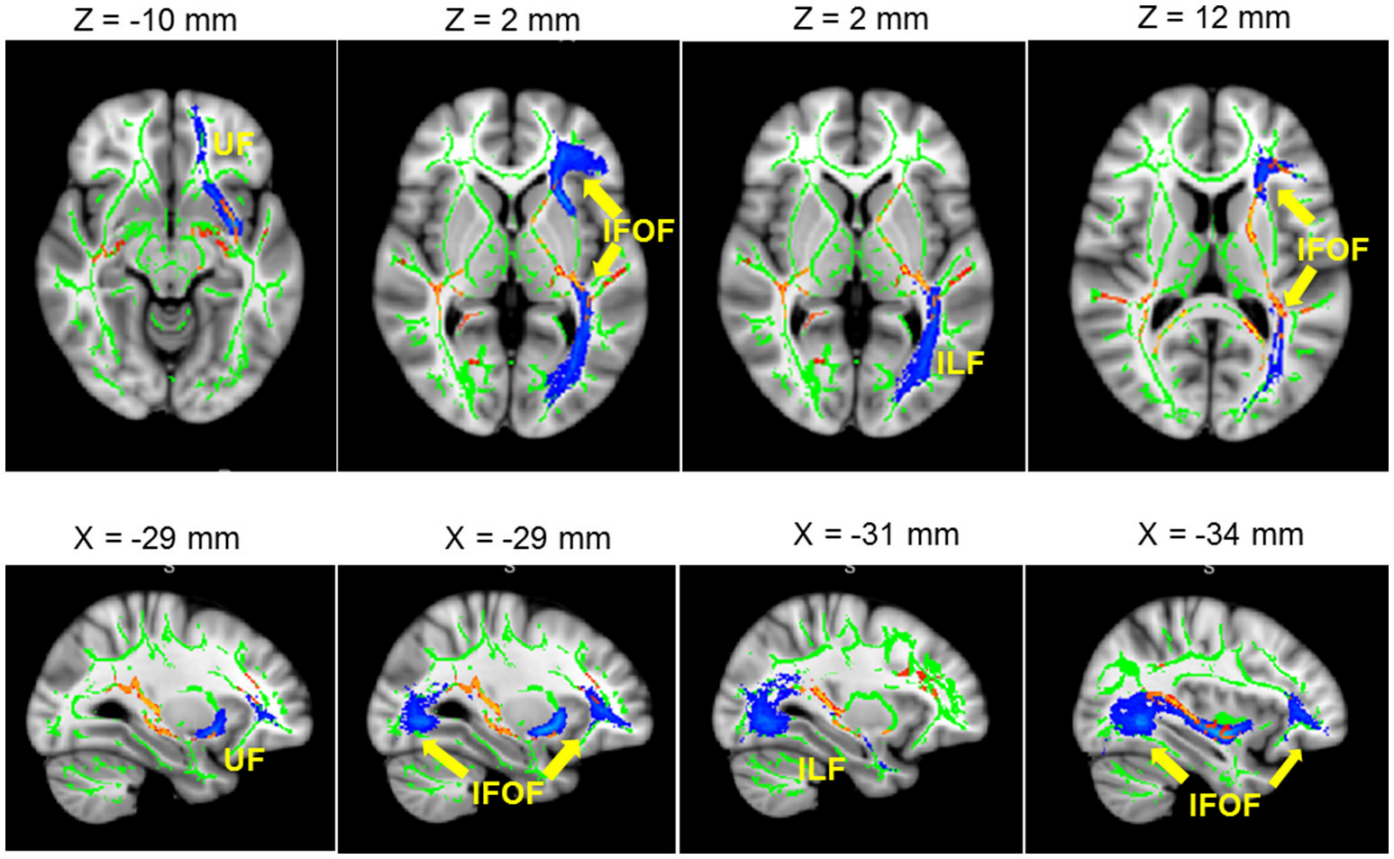
Significant L2 > Monolingual differences in FA values expressed in 1 – *P*-values (*P* < 0.05, corrected, in red) and overlaid onto a standard space and mean FA skeleton (green). Highlighted in blue, some the WM tracts of interest: from left to right. UF, IFOF, and ILF. Higher panel: axial plane. Lower pane: sagittal plane.

The results showed a significant difference in FA between L2 learners and monolingual speakers in a broad network of WM tracts (*p* < 0.05, corrected). Table 2 and Figure 2 present the FA results from the group comparisons between the L2 group and the monolingual group. Sliced were selected each 5 mms and representative voxels were identified. For each WM cluster with significantly larger FA values for the L2 learners group, we report one representative voxel location in MNI152 standard space. Higher FA values were found for the L2 group in the anterior-posterior corona radiata, extending ventrally to the anterior and the retrolenticular portion of the internal capsule, up to the posterior thalamic radiation. More specifically, higher FA values for L2 learners were observed in the anterior and posterior corona radiata which represent a network of fibers that weaves through the internal capsule and that crosses with the fibers of the corpus callosum (CC), including WM tracts of the ATR, the inferior fronto-occipital fasciculus (IFOF), and the uncinate fasciculus (UF). Moreover, higher FA values within the ATR continued ventrally into the anterior limb. Greater FA values in L2 learners were found in the IFOF, ATR, and within bundles of the Inferior Longitudinal Fasciculus (ILF). Finally, greater FA values for L2 learners were found in the posterior thalamic radiation which has connections to ILF and IFOF WM tracts. Monolinguals did not show significantly higher FA values than bilinguals in any region.

**Table 2:** WM clusters with significantly larger FA values for the L2 learners group.

**x, y, z (Representative voxel)**	**Hem**.	**Brain area**	**WM tracts**	***p*-value**
−22, +18, +17	Left	Anterior Corona Radiata	ATR IFOF UF	*p* = 0.042
−27, −43, 22	Left	Posterior Corona Radiata	ATR IFOF	*p* = 0.032
−19, +4, +12 −19, +17, +2	Left	Internal Capsule: anterior limb	ATR	*p* = 0.042 *p* = 0.042
−32, −30, +7	Left	Internal Capsule: retrolenticular portion	IFOF	*p* = 0.034
−33, −27, +2	Left	Internal Capsule: retrolenticular portion	IFOF ILF ATR	*p* = 0.034
−33, −39, +12	Left	Posterior Thalamic Radiation	IFOF ILF	*p* = 0.032
38, −32, +2	Right	Internal Capsule: retrolenticular portion	IFOF ILF	*p* = 0.042
36, −33, +7	Right	Retrolenticular part of the internal capsule	IFOF ILF	*p* = 0.040
33, −34, +12	Right	Internal Capsule: retrolenticular portion	IFOF	*p* = 0.042

*For each cluster, we report one representative voxel location in MNI space. ATR, Anterior thalamic radiation; IFOF, Inferior fronto-occipital fasciculus; UF, Uncinate Fasciculus; ILF, Inferior longitudinal fasciculus*.

**Figure 3:**
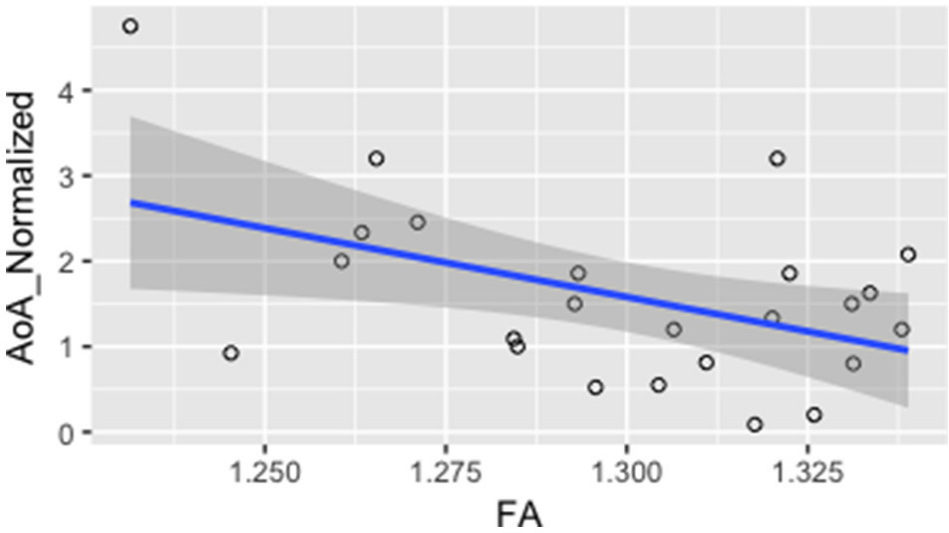
Correlation between mean FA and L2 normalized AoA *r* = −0.465; *p* = 0.02.

In order to investigate whether FA values were correlated with measures of L2 language acquisition, proficiency, and length of immersion in the L2 environment, the mean FA from the voxels showing a significant difference between monolinguals and bilinguals was correlated with measures of, *L2 AoA, L2 proficiency* measured independently through the DELE grammar score, self-proficiency reports, a naming task, and a proficiency composite score (see Methods section for details), and *L2 immersion* measured in months. Results showed a significant correlation between FA and AoA (*r* = −0.46; *p* = 0.02), and between FA values and a normalized index of AoA (AoA/years of speaking the L2; *r* = −0.465; *p* = 0.02) which was calculated to normalize AOA values relative to the number of years participants had been speaking Spanish due to variation in the age of the participants (Figure 3). There were no significant correlations or trends between FA and proficiency, or FA and length of immersion in the L2 (*r* = −0.21; *p* = 0.31). Note that given that not all participants mentioned when they returned to the US from their study abroad experience, we do not have a precise metric to calculate time in the L1 environment after immersion in the L2. However, from the available data the minimum time elapsed from returning to testing was 4 months. For all the remaining participants (who provided that information) they all returned to the US more than 1 year before testing. Additionally, the correlation analysis was run excluding participants who did not report the length of their stay abroad in the language history questionnaire. We reasoned that part of why we fail to find a significant effect is that the distribution of the amount of time spent abroad was not sufficient to show an effect. Even though there was variability in the number of months spent abroad, there was a significant portion of participants who did not study abroad.

## Discussion and conclusions

The goal of this study was to investigate structural changes in WM related to L2 acquisition, especially when the L2 is acquired relatively later in life after a putative sensitive period for L2 learning (Long, 1990; Birdsong, 1999). We also asked whether observed changes were modulated by factors such as AoA, proficiency, and language-use measures such as length of immersion in the L2 environment. WM fractional anisotropy was analyzed using TBSS and results were compared between a group of English-speaking, late L2 learners of Spanish and a group of monolingual English speakers.

The results revealed differences in WM FA between the two groups. L2 learners showed higher FA values in a number of WM tracts in the left hemisphere, including WM tracts of the ATR, the IFOF, the uncinate fasciculus (UF), and the ILF. These results are in line with a number of studies that have reported adaptive WM changes in similar tracts in early (e.g., Mohades et al., 2012, 2015) and late bilingualism (e.g., Pliatsikas et al., 2015, 2017). The data we report supports the growing body of literature proposing that bilingualism and L2 learning promote not only functional but also structural neural adaptation (Li et al., 2014; Bialystok, 2017). Similar to the research conducted by Pliatsikas and colleagues (Pliatsikas et al., 2015, 2017), our study examined the effects of L2 learning on WM structure in adult speakers who learned the L2 later in life (average AoA = 12.1) and were therefore not early bilinguals. However, unlike the speakers in Pliatsikas et al. (2015, 2017), the L2 learners in our study were not immersed in their L2 environment at testing, but were immersed in their native language environment (English). This factor may account for some of the differences observed between our results and Pliatsikas et al.'s results. We will further discuss potential reasons for some of the observed differences between our results and Pliatsikas et al.'s results.

The results revealed higher FA values for L2 learners in the anterior and posterior corona radiata. This WM tract has been previously found to be part of a network implicated during simultaneous interpretation (Hervais-Adelman et al., 2014), and lesions in this region have been shown to lead to word retrieval problems in productive aphasia (Schnur et al., 2006) suggesting its importance for lexical retrieval. The present data are therefore in line with previous results on the recruitment of this WM tract during high-performance bilingual language processing (Hervais-Adelman et al., 2014). Our findings moreover reveal that this WM fiber tract is also implicated in lower performing bilinguals, suggesting that L2 learning also leads to neuro-adaptive changes in WM tracts that are at play during high-performing bilinguals, such as in simultaneous interpreters. The corona radiata is also one of the WM regions that have shown a consistent decline in FA in non-pathological aging (Kennedy and Raz, 2009). As such, these data also suggest that even late bilingualism may contribute increase neural connectivity in non-pathological aging (Kennedy and Raz, 2009), as well as in pathological brain decline (e.g., Luk et al., 2011; Abutalebi et al., 2015b; Perani and Abutalebi, 2015). Late L2 learning may strengthen neural pathways, including those that are most sensitive to age-related neural decline.

Across all of the identified WM regions, greater FA values in L2 learners were observed within the IFOF extending ventrally into the internal capsule, and the posterior thalamic radiation. According to recent language neural models, the IFOF represents a large ventral pathway implicated in language processing that connects the inferior frontal gyrus (BA45/47) with the superior temporal gyrus (BA22), the inferior parietal cortex (BA39), and the occipital cortex; all have been implicated in language comprehension and have been proposed to be central to semantics and spoken word recognition (Leclercq et al., 2010), and semantic processing in general (Duffau, 2008; Martino et al., 2010; see López-Barroso et al., 2013 for null results). Crucially, similar FA increase in the IFOF has been reported in a number of bilingual studies including in children (Mohades et al., 2012, 2015), younger adults (Pliatsikas et al., 2015), and in older bilingual adults (e.g., Luk et al., 2011; Gold et al., 2013 for null results FA values in IFOF in older adults), suggesting that bilingualism might contribute to boosting neural reserve, that might be accumulated throughout the life-span. The present results contribute to the evidence that bilingualism modulates the WM pathways implicated in language processing.

These data also showed higher FA values for L2 learners in the ATR, a bundle of fibers that weaves through the left anterior corona radiata and the anterior and retrolenticular limb of the internal capsule. ATR is a WM tract implicated in lexico-semantic processing by connecting a distributed language network in temporal, parietal, and frontal cortices (e.g., Han et al., 2013; Mirman et al., 2015). Disruption of ATR gives rise to semantic progressive primary aphasia (Han et al., 2013), and auditory verbal hallucinations (AVH) in schizophrenia are negatively correlated with FA values in the ATR (Curčić-Blake et al., 2015), highlighting its role during speech processing (Curčić-Blake et al., 2012). Although the literature has reported some null effects for this WM tract for bilinguals (e.g., Cummine and Boliek, 2013) significant differences within the ATR have been reported in older bilingual adults (Luk et al., 2011) and in children (Mohades et al., 2012). Higher FA values in bilateral ATR have also been found in Spanish-English bilinguals who emigrated to the US in adulthood (Kuhl et al., 2016), and who learned English later in life (mean age = 19.4 years; range = 4.5–28.5 years), and who were immersed in their L2 environment at testing. Additionally, Kuhl and colleagues also found a positive correlation between FA values in the ATR and years of immersion in the L2, and speaking abilities, suggesting that the degree of neural restructuring in ATR was proportional to L2 language experience. Overall, the present data corroborate previous results in showing higher FA values in bilinguals (early and late) for WM tracts that connect a distributed language network.

Finally, in line with our predictions, the results demonstrate higher FA values for L2 learners in the Uncinate Fasciculus, which connects inferior frontal and anterior temporal regions, and more ventrally in the ILF which connects anterior and posterior temporal regions. Although, the past literature is not conclusive regarding WM changes in the UF in bilingualism (García-Pentón et al., 2014; Grundy et al., 2017 for a recent review), the results we have reported are in line with a number of previous studies that have reported higher FA values in the UF in young late L2 learners (Pliatsikas et al., 2015), and in older bilingual adults (Luk et al., 2011). Anterior WM pathways such as the UF are involved in language production (Roelofs, 2014), aspects of syntactic processing (Friederici et al., 2006; Duffau et al., 2009; but see Teichmann et al., 2015 for a counter proposal), new word learning and consolidation (López-Barroso et al., 2013; Ripollés et al., 2014), semantic performance in healthy older adults (de Zubicaray et al., 2011), and primary progressive aphasia (Han et al., 2013). The current results suggest that bilingualism and L2 learning enhance the utilization of those pathways to process both the L1 and the L2, possibly leading to higher neural integrity. Similarly, the ILF connects a ventral language network within temporal and posterior occipitotemporal regions to inferior frontal regions via the UF (Anwander et al., 2007). Consistent with our results, a number of WM connectivity studies have identified changes in the ILF in bilinguals (e.g., Hosoda et al., 2013), which have also been positively correlated with AoA (Nichols and Joanisse, 2016), supporting the idea that ILF pathways are strengthened through L2 processing.

Taken together, the data we have reported support the predictions based on previous WM studies in bilingualism, strengthening the general view that learning, and mastering a second language, even later in life, results in neuroplastic changes. Unlike past studies, the present study did not show a main effect in the corpus callosum (CC). Contrary to what we observed, a number of studies on WM across the life-span have reported effects of bilingualism on the CC (e.g., Luk et al., 2011; Schlegel et al., 2012; García-Pentón et al., 2015; Pliatsikas et al., 2015; but see also Cummine and Boliek, 2013, a study which reports no effects in the CC). One possible factor that might contribute to the absence of a clear effect in CC is our population, which included participants who were immersed in the L1, English, and not in the L2. It is also plausible to think that the absence of changes in CC in our data could be related to the acquisition of the L2 through more formal L2 instruction, rather than through pure immersion. Even though it is plausible to think that the bilingual speakers tested in Pliatsikas et al.'s (2015) did learn their L2 also through some formal learning either before moving to the UK, or while in the UK, they were immersed in the L2 environment at testing. Similarly, most of the studies that report effects in the CC tested participants who were either immersed in their L2 (e.g., Luk et al., 2011; Pliatsikas et al., 2015), or who were engaged in an intensive L2 language training (Schlegel et al., 2012). Again, because our sample of L2 speakers was immersed in their L1 environment, the reported results might, if anything, provide an underestimation of the effects that might be observed under conditions of immersion in the L2 (as for the sample of bilinguals in Pliatsikas et al., 2015). Moreover, our sample was more homogenous relative to Pliatsikas et al.'s relative to the L1. The speakers in the present study were all native speakers of English while Pliatsikas et al.'s bilinguals L1 language background varied. It could be hypothesized that variability in the L1, and linguistic distance between the L1 and the L2 may play a role in engaging neural pathways differentially. Future studies will need to address the question of how immersion in different linguistic environments, and variability in linguistic properties between the L1 and the L2 might modulate these effects.

An additional goal of this research was to understand how WM changes might be correlated with different measures of L2 learning and experience, including AoA, L2 proficiency levels, and length of immersion in the L2. First, the results showed a correlation between FA values and AoA, in line with previous studies on simultaneous and sequential bilingual children (Mohades et al., 2012, 2015). The present data demonstrate that structural changes occur as a consequence of L2 learning, even when the L2 is acquired past childhood. Our data therefore demonstrate that L2 learning promotes neural restructuring. What is novel about these results is the observation that AoA might still be an important factor to explain variation in the observed structural changes, even when the L2 is learned past childhood. However, rather than reasoning in terms of AoA, which is a non-dynamic measure, it is tempting to propose that AoA should be rather interpreted more as a dynamic measure that could encompass length of time spent engaging in L2 learning.

The results did not reveal any correlation between mean FA values and proficiency in the L2. Previous results on the effects of proficiency on structural changes in L2 learners are at best mixed, and do not yet provide a clear picture of how variability in L2 proficiency affects structural changes. While some research has highlighted how structural changes can be correlated with increasing proficiency, and rate of successful L2 learning (e.g., Schlegel et al., 2012; Stein et al., 2012; Hosoda et al., 2013), other studies have failed to find a significant correlation between WM changes and proficiency (e.g., Stein et al., 2014). A number of factors could play a role in the lack of a correlation between structural csdhanges and proficiency across studies. First, proficiency is often measured in different ways across studies, and most importantly, those measures rely at times on subjective measures of proficiency only, and not on objective measures of performance. Additionally, AoA and proficiency are often highly conflated, preventing clear identification of their relative contributions. Finally, some of the studies that report a significant positive effect of proficiency on WM plasticity are training paradigms. For example, Hosoda et al. (2013) exposed participants to an intensive vocabulary learning regime, and they report that after intensive training changes in WM pathways such as the IFGop-caudate and IFGop-STG/SMG pathways were positively correlated with learning success. In this case however, it could be argued that changes in proficiency levels are an outcome by itself, rather than a predictor.

The participants tested in our study were not necessarily exposed to active L2 training. They were intermediate learners of Spanish who were recruited based on L2 Spanish knowledge, but who might or might have not been actively involved in Spanish learning at the moment of testing. We reason that the fact that there was variability in terms active exposure to L2 at testing might have contributed to the absence of correlation between FA and proficiency. Moreover, the variability in their L2 proficiency was pretty limited (mean accuracy on the picture naming in Spanish = 0.55; *SD* = 0.22), and this factor might also have undermined the likelihood to find a significant correlation between FA and L2 proficiency. Overall, future research should address variability in how proficiency is measured. More similar measures of L2 proficiency across studies would allow to create a common basis for measuring and comparing L2 proficiency across studies.

Evidence on the role of immersion in a naturalistic L2 environment as a catalyst for neural change is still mixed (Stein et al., 2014; Pliatsikas and Chondrogianni, 2015). Changes in GM have been reported in simultaneous bilinguals (Burgaleta et al., 2016), in relatively early sequential trilinguals (Abutalebi et al., 2013b), and in later bilinguals (Zou et al., 2012a; DeLuca and Pliatsikas, 2017; Pliatsikas et al., 2017), and WM changes have been observed in immersed L2 learners in left inferior frontal-occipital fasciculus (L-IFOF) and SLF (Stein et al., 2014). The few studies that tested L2 learners who immersed themselves in the L2 environment past childhood mostly fail to observe a correlation between FA and length of immersion (Pliatsikas et al., 2015; DeLuca and Pliatsikas, 2017). Interestingly, a recent reanalysis of Pliatsikas et al.'s (2015) data using diffusion MRI connectometry and correlation analysis (Rahmani et al., 2017) revealed increased connectivity in corpus callosum (CC), arcuate fasciculus (AF), and left IFOF of sequential bilingual adults, and reported positive association with language immersion period, and showed that FA of all of the significant fibers from connectometry analysis, had direct correlation with the duration of immersion period of bilinguals. Our TBSS data does not show any correlation between FA and length of immersion in the L2 environment. One possible explanation for the absence of an effect is the small variability in length of immersion in the L2 (mean length in months: 6.64; range = 0–24), and the fact that all the L2 learners were immersed in their L1 environment at testing (contra Cummine and Boliek, 2013; Kuhl et al., 2016; DeLuca and Pliatsikas, 2017). Cummine and Boliek's (2013) study is likely to be the one with the most similar participants characteristics to ours. They tested adult Chinese–English bilinguals (mean age, 24.2; L2 AoA before the age of 5) and 11 English monolinguals (mean age, 28.5). Crucially however, in Cummine and Boliek, bilingual participants were immersed in the L2 (English), and no specific analyses were reported to correlate those results with length of immersion in the L2 environment. Our participants instead were immersed in the L1 environment at time of testing, and time since returning from being immersed in an L2 environment varied extensively. These factors might account for differences between the results reported by Cummine and Boliek and our results. In sum, given the relatively scarce literature on the potential neuroplastic effects of L2 immersion in late L2 bilinguals, a clear conclusion about the role of immersion will await future research.

To conclude, the present study reveals that L2 learning has the potential to shape the WM networks underlying language processing, even when the L2 is learned after childhood. Given the growing literature suggesting that L2 learning, and long-life experience with the L2 can lead to cognitive, and neural changes which might confer cognitive protection in healthy and pathological aging (inter alia Luk et al., 2011; Alladi et al., 2013; Gold et al., 2013; Bak et al., 2014a,b; Grady et al., 2015; Olsen et al., 2015), it is tempting to proposes that learning a second language throughout the life-span, even during adulthood should become one experiential form of continuous learning available to everyone.

## Ethics statement

This study was carried out in accordance with the recommendations of the PSU IRB board with written informed consent from all subjects. All subjects gave written informed consent in accordance with the Declaration of Helsinki. The protocol was approved by the PSU IRB committee

## Author contributions

ER: Designed the task, collected the data, contributed to the data analysis, and wrote the manuscript; HC: Performed the data analysis, and contributed to the preparation of the manuscript; JK: Designed the task, and contributed to the preparation of the manuscript; MD: Contributed to design the task, contributed to collected the data, and contributed to the preparation of the manuscript; SN: Contributed to collected the data, contributed to the data analysis, and contributed to the preparation of the manuscript.

### Conflict of interest statement

The authors declare that the research was conducted in the absence of any commercial or financial relationships that could be construed as a potential conflict of interest.

## Appendix a: language history questionnaire

Language History Questionnaire

This questionnaire is designed to give us a better understanding of your experience with other languages. We ask that you be as accurate as thorough as possible when answering the following questions.

1. Gender
  ❏ Male
  ❏ Female
2. Age:
3. Do you have any known visual or hearing problems (corrected or uncorrected)?
  ❏ Yes
  ❏ No
4. Native Country
  ❏ United States
  ❏ Other ———————————————————
5. Native Language
  ❏ English
  ❏ Other ———————————————————
6. Language(s) spoken at home (Please check all that apply).
  ❏ English
  ❏ Spanish
  ❏ German
  ❏ Other [Please explain: ———————————————–
  If ENGLISH is your Native Language, please RATE yourself:
  ^***^If English is NOT your Native Language, please contact Experimenter for further instructions.

7. Please rate your English reading proficiency. (1=not literate and 10 = very literate)

**Table:**

	❏ 1 ❏ 2 ❏ 3 ❏ 4 ❏ 5	❏ 6 ❏ 7 ❏ 8 ❏ 9 ❏ 10	

8. Please rate your English writing proficiency. (1=not literate and 10=very literate)

**Table:**

	❏ 1 ❏ 2 ❏ 3 ❏ 4 ❏ 5	❏ 6 ❏ 7 ❏ 8 ❏ 9 ❏ 10	

9. Please rate your English speaking ability. (1=not fluent and 10=very fluent)

**Table:**

	❏ 1 ❏ 2 ❏ 3 ❏ 4 ❏ 5	❏ 6 ❏ 7 ❏ 8 ❏ 9 ❏ 10	

10. Please rate your English speech comprehension ability. (1=unable to understand conversation and 10=perfectly able to understand)

**Table:**

	❏ 1 ❏ 2 ❏ 3 ❏ 4 ❏ 5	❏ 6 ❏ 7 ❏ 8 ❏ 9 ❏ 10

**The next section of the questionnaire deals with your second language learning experience**.

11. Have you studied any second language?
  ❏ No **If NO, please go to question #19**
  ❏ Yes
    **If YES, where and when?** Please check all that apply and indicate length of study.
    ❏ Home Language: ———————————————
    ❏ Since Age ( )
  Elementary School Language: ————————————
  ❏ ( ) year(s)
  Middle School Language: — ——————————————
  ❏ ( ) year(s)
  High School Language: — ——————————————
  ❏ 1 year
  ❏ 2 years
  ❏ 3 years
  College Language:
  ❏ Have not studied a second language in college
  ❏ 1-2 semesters
  ❏ 3-4 semesters
  ❏ 5-6 semesters
  ❏ 8+ semesters
12. If you are taking/have taken any second language at college, please answer the following question. Are you: (Please check all that apply.)
  ❏ Taking a second language for a requirement but interested in being a major or minor.
  ❏ A second language minor
  ❏ A second language major
  ❏ A second language graduate student
  ❏ Other [please explain ——————————————-]

13. Have you studied / lived abroad?
  ❏ Yes
  ❏ No
  **If Yes**, where and when did you study, for how long, and what language did you speak?

**Table:**

Country	Approx. dates	Length of Stay	Language
			
			

**The next section asks you to rate your skills in your primary second language**.

14. Please rate your second language reading proficiency. (1=not literate and 10=very literate)

**Table:**

	❏ 1 ❏ 2 ❏ 3 ❏ 4 ❏ 5	❏ 6 ❏ 7 ❏ 8 ❏ 9 ❏ 10

15. Please rate your second language writing proficiency. (1=not literate and 10=very literate)

**Table:**

	❏ 1 ❏ 2 ❏ 3 ❏ 4 ❏ 5	❏ 6 ❏ 7 ❏ 8 ❏ 9 ❏ 10	

16. Please rate your second language speaking ability. (1=not fluent and 10=very fluent)

**Table:**

	❏ 1 ❏ 2 ❏ 3 ❏ 4 ❏ 5	❏ 6 ❏ 7 ❏ 8 ❏ 9 ❏ 10	

17. Please rate your second language speech comprehension ability. (1=unable to understand conversation and 10=perfectly able to understand)

**Table:**

	❏ 1 ❏ 2 ❏ 3 ❏ 4 ❏ 5	❏ 6 ❏ 7 ❏ 8 ❏ 9 ❏ 10

18. In my second language class es I get:
  ❏ Mostly A's
  ❏ Mostly A's and B's
  ❏ Mostly B's
  ❏ Mostly B's and C's
  ❏ Mostly C's
19. If you speak or have studied more than one second language, please explain about your additional language experience (i.e., years, level of proficiency, etc.)

___________________________________________________________________________________________________

Thank you for your participation
